# Long intergenic non-protein-coding RNA 1567 (LINC01567) acts as a “sponge” against microRNA-93 in regulating the proliferation and tumorigenesis of human colon cancer stem cells

**DOI:** 10.1186/s12885-017-3731-5

**Published:** 2017-11-06

**Authors:** Xiaofeng Yu, Lin Mi, Jie Dong, Jian Zou

**Affiliations:** 1 Department of Gastroenterology, Huadong Hospital Affiliated to Fudan University, West Yan’an Road 221, Shanghai, 200040 China; 2 Drug Clinical Trial Organization Office, Huadong Hospital Affiliated to Fudan University, Shanghai, 200040 China

**Keywords:** LINC01567, MicroRNA-93, Colon Cancer stem cells, Regulation

## Abstract

**Background:**

Cancer stem cells (CSCs) are considered to be the major factor in tumor initiation, progression, metastasis, recurrence and chemoresistance. Maintaining the stemness and promoting differentiation of these cells involve various factors. Recently, long non-coding RNAs (lncRNAs) have been identified as new regulatory factors in human cancer cells. However, the function of lncRNAs in colon CSCs is still unknown.

**Methods:**

Primary colon cancer cells were maintained in serum-free medium to form spheres and CD133^+^/CD166^+^/CD44^+^ spheroid cells were selected using FACS technique. Then we detected growth curve, colony formation, invasion and migration ability, and tumorigenicity of CD133^+^/CD166^+^/CD44^+^ cells. LOCCS-siRNA and pcDNA-LOCCS plasmid vectors were constructed and transfected to evaluate impact of the lncRNA. We also performed dual luciferase reporter assay to verify the interaction of LOCCS and miR-93.

**Results:**

The research explored lncRNA expression and the regulatory role of novel lncRNAs in colon CSCs. Using the stem cell markers CD133, CD166 and CD44, we found a subpopulation of highly tumorigenic human colon cancer cells. They displayed some characteristics of stem cells, including the ability to proliferate and form colonies, to resist chemotherapeutic drugs, and to produce xenografts in nude mice. We also found an lncRNA, LOCCS, with obviously upregulated expression in colon CSCs. Knockdown of LOCCS reduced cell proliferation, invasion, migration, and generation of tumor xenografts. Furthermore, microRNA-93 (miR-93) and Musashi-1 mediated the tumor suppression of LOCCS knockdown.

**Conclusions:**

There was reciprocal repression between LOCCS and miR-93. Research on mechanisms suggested direct binding, as a predicted miR-93 binding site was identified in LOCCS. This comprehensive analysis of LOCCS in colon CSCs provides insight for elucidating important roles of the lncRNA–microRNA functional network in human colon cancer.

**Electronic supplementary material:**

The online version of this article (10.1186/s12885-017-3731-5) contains supplementary material, which is available to authorized users.

## Background

Colorectal carcinoma (CRC) is in the third of malignant tumor in men and second in women, and in 2008, there were about 1.2 million new patients and 600,000 death cases [1]. Its incidence is still rising owing to aging populations with unhealthy eating habits. Despite efforts to improve clinical treatment, the prognosis of CRC patients has shown no marked progress in recent years. A small group of cells with stem cell properties, has been separated from CRC and they are referred to as CRC stem cells (CR-CSCs) [2]. These cells proliferate infinitely and differentiate into distinct cell types. Although rare in cancer tissue, CR-CSCs play a key role in the maintenance of tumor homeostasis. CSCs are proposed to be the source of malignancy and also the basis of progression, metastasis, recurrence and drug resistance [3, 4]. Therefore, it is important to study the intrinsic mechanisms of CRC maintenance.

In the human genome, there are large amounts of noncoding RNA, including microRNAs (miRNAs) and long noncoding RNAs (lncRNAs, defined as >200 nt). As a new modulator, lncRNAs have gained more and more attention for their roles in stem cell pluripotency, molecular scaffolding, transcriptional gene silencing and maintenance of DNA methylation/demethylation [5–8]. Many researchers have found that lncRNAs are dysregulated in various tumors, although their roles in tumor progression remain unknown [9–11]. LncRNAs are also key modulators of gene expression in stem cells and during carcinogenesis [12, 13]. In addition, miRNAs have been reported to affect CRC tumorigenesis [14, 15]. LncRNAs have the ability to competitively inhibit miRNAs, and act as molecular “sponge”. However, it remains unknown whether lncRNAs affect CRC progression by regulating miRNAs.

We previously isolated and characterized CR-CSCs from the cell line SW1116 (SW1116csc). Using miRNA arrays, we found 46 dysregulated miRNAs in SW1116csc cells in comparison with differentiated SW1116 cells. Among these miRNAs, 35 were overexpressed more than 1.5-fold, and 11 were downregulated. There was a 16.7 fold drop of miR-93 expression in SW1116csc, and the growth and coloning efficiency of SW1116csc were obviously inhibited by elevated expression of miR-93 [16]. However, lncRNAs that may competitively regulate miR-93 in CR-CSCs have not yet been identified.

## Methods

### Patient sample preparation

Tumorous colon tissues and corresponding adjacent non-tumoral colon tissue were collected from ten patients undergoing colon cancer surgery at Huadong Hospital, Shanghai, China. Written informed consents were obtained from all patients. Our protocol was approved by the Clinical Research Ethics Committee of Huadong Hospital. Clinicopathologic features of the ten colon cancer patients, including age, gender, and tumor site, stage, type and differentiation, are listed in Table 1.

**Table 1:** Characteristics of the ten colon cancer patients participating in the present study and the tumor sample information

Case	Gender	Age ranges (year)	Tumor site	Tumor stage	Tumor type	Differentiation
1	M	50–60	CS	I	AC	Well
2	M	70–80	CS	IIIc	AC	Moderately
3	F	40–50	CA	IIa	AC	Poorly
4	M	60–70	CS	IIIb	AC	Poorly
5	F	60–70	CA	I	AC	Moderately
6	F	50–60	CA	IV	AC	Well
7	F	50–60	CS	IIa	AC	Moderately
8	M	80–90	CA	IIIc	AC	Well
9	M	60–70	CS	I	AC	Well
10	F	30–40	CA	IV	AC	Poorly

*CS* colon sigmoideum, *CA* colon ascendens, *AC* Adenocarcinomas

### Primary cultures

After washing with phosphate-buffered saline (PBS), colon samples were minced into 1.0 mm^3^ fragments and dissociated enzymatically with 0.25% trypsin–EDTA (0.53 mM). Tumor/tissue fragments were incubated at 37 °C with pre-warmed enzyme for 100 min. The cell suspension was then filtered and washed with SSM. After dissociation, the cells were purified using Ficoll-Hypaque density centrifugation. Finally, the recovered cell population was washed and resuspended in SSM and antibiotics (penicillin G 100 IU/mL, streptomycin 100 mg/L, metronidazole 1 mg/L, amphotericin B 2.5 mg/L, gentamicin 20 mg/L) (Yihe Biological). Primary cells were seeded into 96-hole plates (10,000 cells/hole) and cultured at 37 °C and 5% CO_2_ for 10 days.

### Culture of colon cancer spheres

The serum-supplemented medium (SSM) contained RPMI 1640 medium and fetal bovine serum (10% final concentration). Serum-free medium (SFM) consisted of DMEM/F12 (HyClone) supplemented with B27 (1:50; Gibco), 20 μg/L EGF (PeproTech), 10 μg/L bFGF (PeproTech), 10 μg/L LIF (Chemicon), 2 mM L-glutamine, 4 U/L insulin, 100 IU/mL penicillin G, and 100 mg/L streptomycin. Primary cultured colon cancer cells from surgery samples were digested with trypsin (Amresco) after washing with PBS and then cultured in SFM. After colon cancer spheres were generated, they were collected by centrifugation at 800 rpm, mechanically dissociated and cultured for progeny cell spheres.

### Flow cytometry

Cell spheroids and normal primary cells were digested using trypsin and resuspended in PBS (5 × 10^6^/mL). Cells were incubated with FITC-conjugated anti-CD44 and PE-conjugated anti-CD133/CD166 monoclonal antibodies at 4 °C (30 min). The percentage of positive tumor cells was calculated by detection of fluorescence intensity of the molecules (CD44, CD133 and CD166). The FC500 flow cytometer from Beckman Coulter was used to analyze the samples.

### Western blotting

Cells were added with lysing buffer consisted of 20 mM Tris-HCl, 0.1% (*w*/*v*) Triton X-100, 0.5% sodium deoxycholate, 1 mM phenylmethylsulfonyl fluoride, 10 mg/L leupeptin, and 10 mg/L aprotinin. Then the mixture was centrifuged with 12,000×*g*. BCA assay was used to measure total protein concentration. Protein of extract samples (50 μg) was added to 10% SDS-PAGE following PVDF electrophoresis (Invitrogen). Protein blots were probed with primary antibodies in 5% milk in Tris-buffered saline at 4 °C overnight. The antibodies were against glyceraldehyde-3-phosphate dehydrogenase (GAPDH), Oct-4, Musashi-1 (MSI1), ABCG2, Sox2 and Klf4 (Santa Cruz Biotechnology).

### Cell proliferation and colony formation assays

Spheroid cells and primary cultured cells (1 × 10^4^) were seeded in 24-pore plate. 48 h later, trypan blue (Jianglai Bio) was added and cells were counted in triplicate over six weeks. For colony formation, spheres were digested with trypsin and resuspended in medium containing 0.3% agar. Then the mixture was plated onto a 0.6% agar bottom layer. Each culture dish contained 1 × 10^3^ cells. After 14 days, clones with diameters larger than 0.5 mm were counted.

### Cell invasion and migration assays

The ability of invasion and migration of CD133^+^/CD166^+^/CD44^+^ spheroid cells or primary cultured cells was evaluated using transwell chambers (8 μm pore) polycarbonate membrane (Corning). Cells were seeded above the membrane. Matrigel (Becton Dickinson) was used to cover the top side of membrane for invasion assay and Matrigel-free for the migration assay. After culture at 37 °C for 48 h, cells inside the upper chamber were removed. 95% ethanol was used to fix the migrated and invaded cells under the membrane. After 0.2% crystal violet stained, the cells were counted under a microscope (five fields per well).

### Drug sensitivity assays

Spheroid cells and primary cultured cells (1 × 10^4^ per well) were seeded onto 96-hole plates containing different concentrations of chemotherapeutic drugs or PBS. After 48 h, Alamar Blue dye (Invitrogen, USA) was added in amounts equal to 10% of medium volume and cultured for 4 h. Then absorbance of the mixture were measured using a microplate reader (Bio-Rad, Model 550) at 570 nm and 600 nm.

### Establishment of tumor xenografts in nude mice

For animal experiment, 6 week old female nude mice were used, which from the Weitong Lihua Laboratory Animal Center (Beijing, China). Mice were fed for one week in a specific pathogen-free animal cage before intervention. CD133^+^/CD166^+^/CD44^+^ spheroid cells were selected using flow cytometry as the experimental group. The primary cultured cells served as a control. Cells (1 × 10^5^) were injected subcutaneously in the right flank of each mouse and observed the tumorigenicity. In the plasmid transfection assay, three groups of mice were injected with CD133^+^/CD166^+^/CD44^+^ cells containing different plasmids. Tumors were measured weekly using electronic calipers. The volume of tumor was calculated using the formula V = (4/3)πxy^2^. x is the half of the longest diameter (a) and y is half of the perpendicular axis (b). The mice were sacrificed 63 days after inoculation, and 10% neutral formalin was used to fix the tumors. All animal experiments were approved by the Institutional Committee for Animal Research and followed the national guidelines for the care and use of laboratory animals (GB14925–2010).

### Real-time quantitative reverse transcription PCR

Total RNA was isolated from cultured cells using the standard TRIzol method. 100 ng total RNA was used to synthesize cDNA with a SuperScript Reverse Transcriptase kit (Invitrogen). For PCR amplification system, 25 μL reaction mixture was used containing 2 μg of cDNA, 1 μL primers and 12.5 μL 2× SYBR Green PCR Master Mix. An ABI Prism 7000 real-time PCR machine (Applied Biosystems) was used for amplificationn reaction. The primer sequences were 5′-TGCTGGGGAAAGGAGATTGG-3′ (sense) and 5′-AGCAGAAGTAAGGCACGAGG-3′ (antisense) for LOCCS. PCR condition was denaturation at 95 °C, and then 40 cycles of 95 °C (15 s) → 60 °C (30 s) → 72 °C (3 s). Threshold cycle (*C*T) method was used to average and compare the real time values. The value of target RNA (2^−ΔΔCT^) is normalized to β-actin expression reference (Δ*C*T). The amount of target in untreated cells was set as 1.0. Experiments were performed in duplicate.

### LOCCS-small interfering RNA (siRNA) plasmid construction and transfection

Three pairs of siRNA primers (Z1, Z2, Z3) targeting human LOCCS were synthesized and purified by Shanghai Haike Corporation. Annealing was performed in a 10 μL reaction mixture including 4.5 μL forward primer (50 μM), 4.5 μL reverse primer (50 μM) and 1 μL annealing buffer at 95 °C for 5 min and decreased to 30 °C gradually (0.1 °C/s). BLOCK-iT U6 RNAi Entry Vector kit (Invitrogen) was used for ligation in a 10 μL reaction volume containing 1 μL annealed primers, 1 μL pENTR/U6 plasmid, 1 μL T4 ligase buffer, 1 μL T4 ligase and 6 μL deionized H_2_O, and the reactions were placed at 16 °C for 2 h. Then, 5 μL of the ligated product was added to 100 μL DH5X cell solution, and the mixture was placed at 4 °C for 10 min, 42 °C for 90 s, and 4 °C for 5 min. After 300 μL Luria-Bertani medium was added, the mixture was shaked at 220 rpm for 1 h. Finally, transformants were transferred to kanamycin-containing plates at 37 °C overnight. Kanamycin-resistant clones were chosen, and the plasmids were isolated using the lyticase method. The inserted sequences in the plasmid were verified by DNA sequencing. Spheroid cells were transfected with 500 ng of each of the three pENTR/U6-siLOCCS plasmids (Z1, Z2, Z3) with Fugene 6 Transfection kit (Roche). The transfected cells were harvested 48 h later, and expression level of miR-93 was mensurated using quantitative PCR.

### Construction of the pGL3M-miR-93 luciferase reporter plasmid

For the luciferase reporter vector construction, the pre-miR93 sequence was synthesized with added XbaI sites by Shanghai Haike Corporation. The sequence was TGCTCGACTCTAGACTGGGGGCTCCAAAGTGCTGTTCGTGCAGGTAGTGTGATTACCCAACCTACTGCTGAGCTAGCACTTCCCGAGCCCCCGGTCTAGAGCTGCTCG. The sequence was then inserted into a vector containing the pGL3 promoter upstream of the firefly luciferase (FLUC) reporter gene (Invitrogen). Sense (F:CTAGACtgggggctccaaagtgctgttcgtgcaggtagtgtgattaccca acctactgctgagctagcacttcccgagcccccggT) and antisense (R:CTAGAccgggggctcgggaagtgctagctcagcagtaggttgggtaa tcacactacctgcacgaacagcactttggagcccccagT) primers were synthesized, and 4.5 μL of each primer (100 μM) and 1 μL annealing buffer were placed at 95 °C for 5 min, then 25 °C for 30 min. A 10 μL solution containing 5 μL annealed primers, 1 μL PGL3-XbaI plasmid, 1 μL T4 ligase buffer, 1 μL T4 ligase and 2 μL deionized H_2_O was placed at 16 °C for 2 h. Then, 5 μL ligated product was added to 100 μL DH5× cell solution. The mixture was placed at 4 °C for 10 min, 42 °C for 90 s, and 4 °C for 5 min. After 300 μL Luria-Bertani medium was added, the mixture was shaked at 220 rpm for 1 h. Finally, transformants were transferred to kanamycin-containing plates at 37 °C overnight. Positive clones were chosen, and the inserted sequence in the plasmid was verified by DNA sequencing.

### Dual luciferase reporter assay

1 × 10^5^ spheroid cells (per hole) were cultured in 24-hole plates. 48 h later, they were cotransfected with the following combinations of plasmids. For endogenous LOCCS analysis (no exogenous LOCCS transfection), A: 400 ng pGL3M-miR-93 + 400 ng pENTR/U6-si-LOCCS +500 ng pRL-CMV; B: 400 ng pGL3M-miR-93 + 500 ng pRL-CMV; C: 400 ng pGL3M + 500 ng pRL-CMV; for exogenous LOCCS analysis, D: 400 ng pGL3M-miR-93 + 400 ng pcDNA-LOCCS +500 ng pRL-CMV; E: 400 ng pGL3M-miR-93 + 400 ng pcDNA-LOCCS-T + 500 ng pRL-CMV; F: 400 ng pGL3M-miR-93 + 500 ng pRL-CMV. The pRL-CMV plasmid was cotransfected and used as a control. It contains a weak promoter region upstream from the Renilla luciferase gene and alone produces low levels of luminescence. The transfected spheroid cells were harvested 24 h later, and the luciferase content in lysed cells was measured using the Promega Dual Luciferase Reporter assay (Madison). FLUC and Renilla luciferase luminescence of the samples were measured in a luminometer (Promega GloMax 20/20 Luminometer). The result was expressed as fold change in cells receiving treatments relative to media control cells.

### Construction of pcDNA-LOCCS plasmid vectors

The whole gene synthesis method was used to synthesize the LOCCS cDNA. For the total 2907 bp, 162 primers were designed and synthesized by Shanghai Haike Corporation, and each primer was then diluted to 10 μM. The primers were combined into groups of 20 (1–20, 19–40, 39–60, 59–80, 79–100, 99–120, 119–140, and 139–162) containing10 μL of each primer. Then, 5 μL of the mixed primer solution was used for PCR amplification. The 50 μL mixture included 5 μL 10× buffer, 2 μL MgSO_4_, 1 μL dNTPs, 5 μL primer mix, 0.2 μL PFU DNA polymerase, and 36.8 μL H_2_O. The conditions for PCR: 95 °C (5 min), 30 cycles of 94 °C (30 s) → 55 °C (30 s) → 72 °C (1 min), and 72 °C (10 min). When the amplification was completed, 2 μL of the product was used for the amplification of eight larger fragments with the corresponding primers (primers 1 and 20, 19 and 40, 39 and 60, 59 and 80, 79 and 100, 99 and 120, 119 and 140, 139 and 162). The 50 μL PCR reaction mixture included 5 μL 10× buffer, 2 μL MgSO_4_, 1 μL dNTPs, 2 μL primers, 0.2 μL PFU DNA polymerase, and 37.8 μL H_2_O. The PCR conditions were as previously. The PCR products were electrophoresed and then extracted from the gel slice using the AP-GX-50 AxyPrep/DNA Gel Extraction kit (Axygen). Finally, the eight larger extracted fragments were combined and 8 μL used as template for a third round of PCR with primers 1 and 162 to synthesize the entire LOCCS cDNA. The PCR products were cloned into the pMD18-T vector (hereafter referred to as the LOCCS-ox plasmid). A 5 μL reaction mixture containing 2 μL PCR product, 0.5 μL pMD18-T vector and 2.5 μL of buffer, was cultured at 16 °C for 4 h. Using electroporation, the plasmids were transfected into super-competent *Escherichia coli* DH5X and then seeded on ampicillin SOB medium. After 24 h, plasmids from four randomly chosen clones were re-isolated for DNA sequencing.

### Site-directed mutagenesis for construction of pcDNA-LOCCS-T plasmid vectors

According to the complimentary sequences with miR-93, mutagenesis primers were designed (F:TGATCTGACATGGGAGGTCGAGGCC; R:CGATGCAACATGAGCCACCGCGCCT) and used, with the pcDNA-LOCCS plasmid as template, for PCR amplification. Then, the pcDNA-LOCCS-T plasmid was constructed using the TaKaRa MutanBEST kit.

### Lentiviral vector construction, production, and cell infection

The human LOCCS, miR-93, and MSI1-specific siRNA sequences were designed and synthesized by Shanghai Haike Corporation. The nonsilencing sequence 5′-TTCTCCGAACGTGTCACGT-3′ was used as a scrambled control. The LOCCS gene sequence is shown in the Additional file 1: S1. Oligonucleotides complementary to these sequences were synthesized and ligated into the pGCSIL-GFP vectors. Then the plasmids were amplified in *E. coli* DH5. For lentivirus generation, Lipofectamine 2000 (Invitrogen) was used to transfect recombinant pGCSIL-GFP, pHelper 1.0 and pHelper 2.0 vectors into 293 T cells. 48 h later, the lentiviral particles were harvested using 50,000 × *g* ultracentrifugation for 2 h, and they are named as Lv-si-LOCCS, Lv-si-miR-93, Lv-si-MSI1 and Lv-si-NC (negative control). For cell infection, CD133^+^/CD166^+^/CD44^+^ spheroid cells were incubated with lentiviruses at 50 MOI for 48 h, and stable clones were selected in the medium contained 10 mg/mL puromycin (Sigma-Aldrich, USA).

### Statistical analysis

All data were statistically analyzed using Student’s *t* test or repeated one-way ANOVA with Dunnett post hoc test (GraphPad Prism 6, CA, USA). In all statistical analysis, *P* value of *<0.05* was considered significant.

## Results

### Primary human colon cancer cultures from fresh tumor tissue and colon cancer spheres formation

**Fig. 1:**
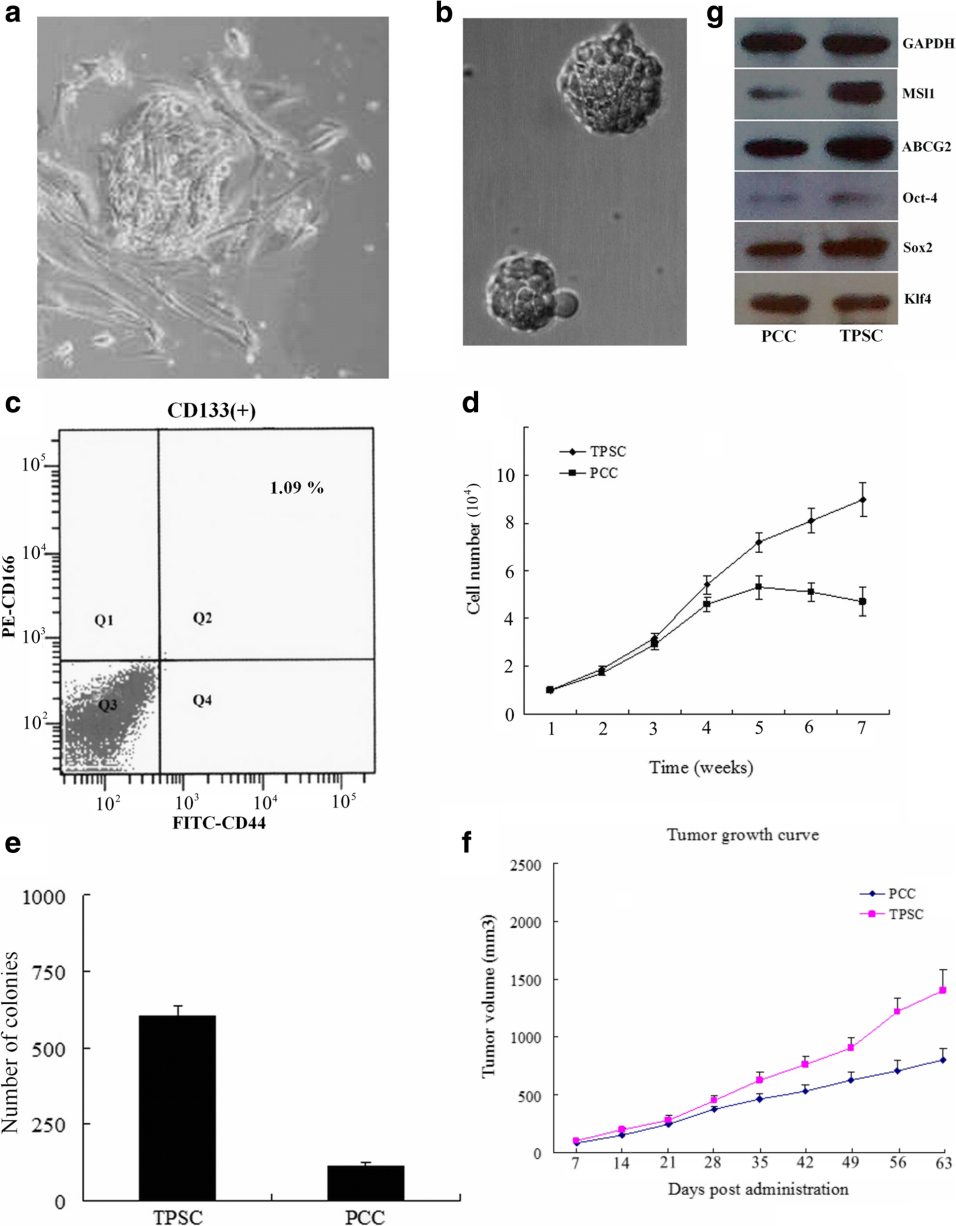
Generation, proliferative capacity, stem-cell markers of TPSC and their tumorigenicity in nude mice. **a** Primary cultured human colon cancer cells under light phase-contrast microscopy (×200). **b** Spheres of human colon cancer cells in the SFM suspension culture system. **c** Expression of CD133, CD166 and CD44 stem cell surface markers in colon cancer spheroid cells. Flow cytometry dot plots showing that colon cancer sphere cells expressed high levels of CD166 and CD44 in SFM. Cancer sphere cells incubated with FITC-conjugated anti-CD44 and PE-conjugated anti-CD166 monoclonal antibodies. **d** Growth curves of TPSC and PCC. **e** Colony formation rates of TPSC and PCC. **f** Tumor xenografts in nude mice. The volume of tumors generated by 1 × 10^5^ TPSC and PCC 63 days after injection. Left: TPSC; Right: PCC. **g** Expression of stem-cell markers in human colon cancer spheroid cells. Each experiment was performed in triplicate

Fresh tumor tissue were digested and cultured in SSM. On the third day, some cells began to attach to the plastic support. After seven days, many cells grew in monolayers attached to the support and some of them began to divide. The primary cultured cells displayed an epithelial morphology, as observed using light microscopy (Fig. 1a). These cultured primary human colon cancer cells were then digested and plated in an SFM suspension culture system. During the initial selection phase, the majority of plated cells died off, and only a few colonies grew out. Spheres were observed on day 6 (Fig. 1b), and they accounted for ~4% of the total number of cells on day 12. The spheres also increased in volume over time.

### Analysis of the expression of surface markers CD133, CD166 and CD44 in primary colon cancer adherent and spheroid populations

CD133, CD166 and CD44 have been reported to isolate CR-CSCs [2, 17, 18]. So we used the three surface markers to detect CR-CSCs in spheroid and also analyzed the expression of them. There was no significant difference in CD133 level between the adherent (8.4%) and spheroid (9.1%) cells (*P* > 0.05). The proportion of CD166^+^ cells in the adherent cells was much smaller (10.2%) than in the spheroid cells (38.5%) (*P* < 0.05). The proportion of CD44^+^ cells in the adherent cells was also much smaller (1.5%) than in the spheroid cells (80.3%) (*P* < 0.05). The CD133 positive cells were further analyzed for parallel expression of CD44 and CD166. A mean of 1.09% of cells were triple positive (CD133/CD166/CD44) (Fig. 1c), and 5.73% (CD133/CD44) and 2.12% (CD133/CD166) were double positive.

### Proliferation and differentiation capacity of colon cancer–derived spheroid cells in vitro and tumor growth in vivo

We assessed the ability of proliferation of these primary cells in SFM. Sphere forming features were found in most primary tumor cells (9 of 10 cultures). These suspended spherical cells were observed within 7 days, and most of them survived in SFM for over 8 weeks. During prolonged propagation, ~5% of cell spheres began to adhere to the plate and formed epithelial morphology with differentiation capacity. When growth factors were removed and the cells were exposed to 10% SSM, most of the cell spheres (>80%) became adherent. As tumor spheres differentiated, cells migrated out and formed monolayer epithelial cells.

We next evaluated the proliferative capacity of CD133^+^/CD166^+^/CD44^+^ spheroid cells (triple positive spheroid cells, TPSC) and found that TPSC in SFM had increased proliferative capacity compared with primary cultured cells (PCC) in SSM (Fig. 1d). The proliferation of colon cancer sphere cells was then assessed using coloning efficiency. TPSC were seeded on 24-hole plates (1000 cells per hole) and produced more numbers of spheres (607 ± 28) than PCC (113 ± 15) (*P* < 0.05) (Fig. 1e).

For checking the tumorigenicity of cell spheroids, transplantation assays were performed and showed that 1 × 10^4^ TPSC were competent to produce tumors, whereas the same number of PCC failed to produce visible tumors within 15 days. The tumor volume generated by 1 × 10^5^ TPSC was significantly greater than that of the control group 63 days after injection (*P* < 0.05), indicating that TPSC have high tumorigenicity (Fig. 1f).

### Expression of stem-cell markers and chemotherapeutic drug resistance in tumor spheroid cells

The expression levels of several stem cell markers (MSI1, Oct-4, Sox2, Klf4 and ABCG2) were detected in TPSC. As shown in Fig. 4, western blot showed that MSI1, Oct-4, Sox2 and ABCG2 had higher expression levels in TPSC than in PCC. However, Klf4 showed no obvious difference in expression levels between TPSC and PCC.

Multidrug resistance of TPSC to paclitaxel, adriamycin, etoposide, cytarabine, fluorouracil, cisplatin and mitomycin was examined in an Alamar blue assay. Compared with PCC, TPSC displayed a marked increase in resistance to these chemotherapeutic drugs. The resistance of TPSC to adriamycin, paclitaxel, mitomycin, etoposide, cisplatin, cytarabine and fluorouracil was 17.4, 13.9, 4.2, 3.0, 2.6, 2.0 and 1.5 folds higher than differentiated cell populations (Table 2). The results show that colon tumor spheroid cells have increased resistance to standard chemotherapy than differentiated cells.

**Table 2:** Sensitivity of colon cancer spheroid cells and primary cultured cells to chemotherapeutic drugs

	IC50 (mg/L)		
Drug	TPSC	PCC	Fold difference
Adriamycin	36.5 ± 2.3**	2.1 ± 0.2	17.4
Paclitaxel	32.0 ± 2.4**	2.3 ± 0.3	13.9
Mitomycin	0.93 ± 0.04**	0.22 ± 0.02	4.2
Etoposide	10.2 ± 0.3**	3.4 ± 0.2	3.0
Cisplatin	10.5 ± 0.6*	4.1 ± 0.3	2.6
Cytarabine	27.7 ± 1.7*	13.9 ± 0.8	2.0
Fluorouracil	50.5 ± 4.1*	33.2 ± 2.2	1.5

**P < 0.01; *P < 0.05; IC50: The half maximal inhibitory concentration

### Expression of a novel lncRNA in colon cancer–derived spheroid cells

**Fig. 2:**
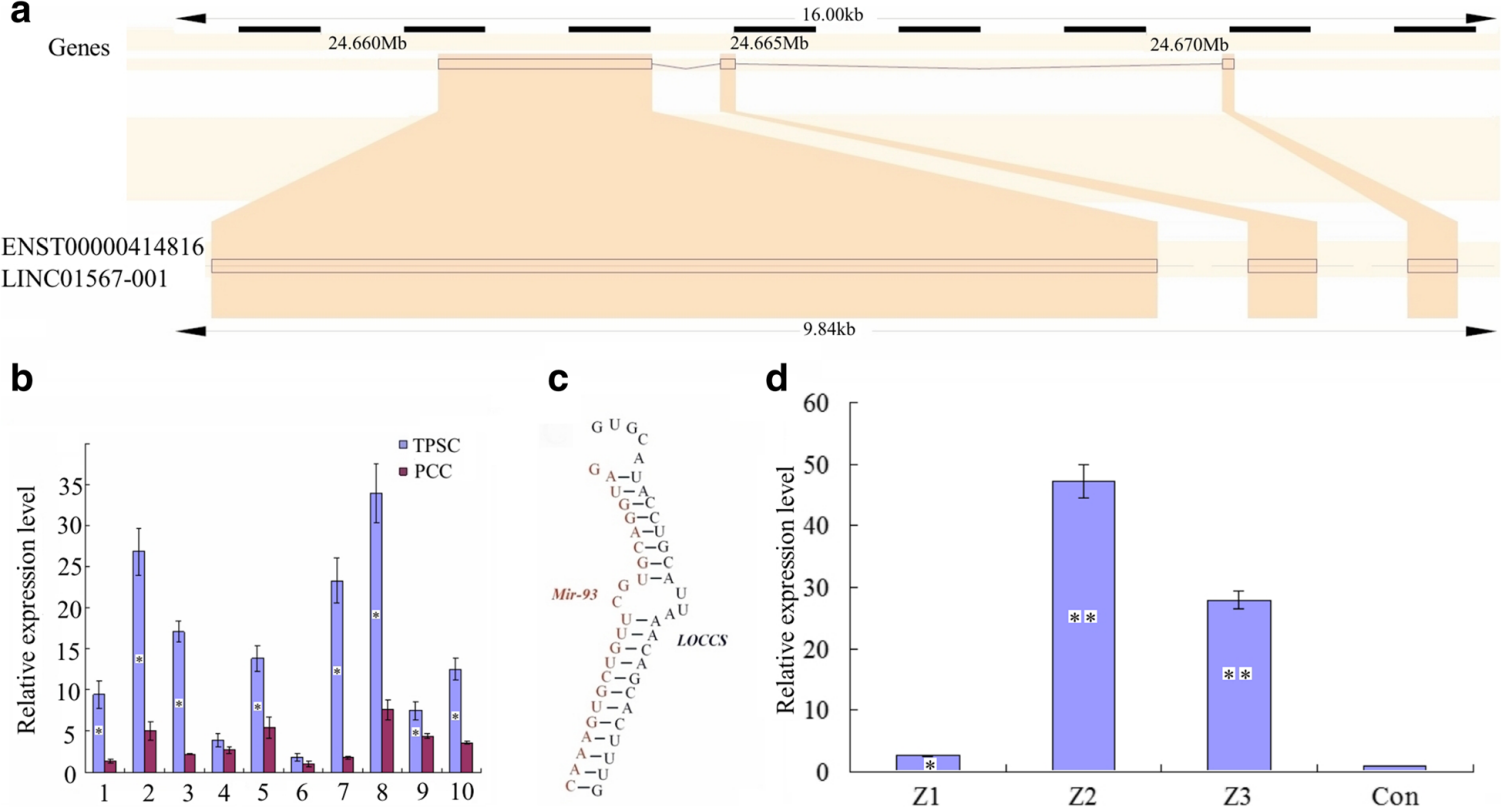
Expression of LINC01567 and its interaction with miR-93. **a** Schematic of the LINC01567 gene and its transcript. **b** Levels of LOCCS in colon cancer spheres and primary colon cancer cells. **c** The predicted binding sites between LOCCS and miR-93. **d** The inhibition ratio of three pENTR/U6-siLOCCS plasmids (Z1, Z2, Z3) of the expression of miR-93. Each experiment was performed in triplicate. ** *P* < 0.01; *P < 0.05

In previous studies, we found the expression of a lncRNA (ENST00000414816, also referred to as long intergenic non-protein-coding RNA 1567; LINC01567) was significantly upregulated in colon cancer–derived spheroid cells (data not published). This lncRNA may play a key role in occurrence and progression of colon cancer. LINC01567 gene is located on chromosome 16 (positions 24,661,422–24,671,062 on the reverse strand), contains three exons and produces one transcript (2907 bp) (Fig. 2a). (sequences in the Additional file 1: S1) We detected the expression levels of LINC01567 (hereafter referred to as LOCCS; lncRNA overexpressed in colon cancer stem cells) in 10 pairs of PCC and TPSC using quantitative PCR. The levels of LOCCS in TPSC were obviously increased relative to PCC (8 in 10 pairs; *P* < 0.05) (Fig. 2b).

### The interaction between LOCCS and miR-93 in CD133+/CD166+/CD44+ spheroid cells

LOCCS was upregulated in TPSC and we concluded that it might play an important role in the proliferation and differentiation of CR-CRCs. Recently, some researchers have revealed that lncRNAs act as miRNA “sponges” to mitigate miRNA activities [19, 20]. Using lncRNA interaction analysis software (Starbase v2.0), we confirmed that LOCCS could bind with miR-93, and the binding region is shown in Fig. 2c. Three pENTR/U6-siLOCCS plasmids (Z1, Z2, and Z3) were constructed and transfected into TPSC to knock down the expression of LOCCS. (sequences in the Additional file 2: S2) Quantitative PCR indicated that miR-93 was upregulated as LOCCS decreased, and the Z2 plasmid was used for the following experiments (Fig. 2d).

To confirm the interaction between LOCCS and miR-93, we synthesized pGL3M-miR-93, pcDNA-LOCCS and pcDNA-LOCCS-T plasmids and transfected them into TPSC. (sequences in the Additional file 2: S2) In the endogenous LOCCS assay, we transfected cells with various combinations of the three plasmids. The expression of the FLUC reporter was inhibited by endogenous LOCCS, as was the expression of miR-93 from pGLM-miR-93. However, when the endogenous LOCCS was degraded by siLOCCS transcribed from the pENTR/U6-siLOCCS plasmid, the inhibition of miR-93 by LOCCS was weakened, and thus FLUC levels were higher (A vs. C, B vs. C; *P* < 0.05) (Table 3). When cells were cotransfected with pcDNA-LOCCS, miR-93 was inhibited by both endogenous and exogenous LOCCS, and, thus, the levels of miR-93 in this group were the lowest among the groups (D vs. F, D vs. E; *P* < 0.05). In contrast, LOCCS-T transcribed from the pcDNA-LOCCS-T plasmid could not combine with miR-93. The miR-93 levels were similar to those of the group without exogenous LOCCS (E vs. F; *P* > 0.05) (Table 3). The observation that if the binding sequence in LOCCS was mutated, it could not combine with miR-93 confirmed that LOCCS acts on miR-93 directly.

**Table 3:** Dual luciferase reporter assays confirm the interaction between endogenous or exogenous LOCCS and miR-93

	Endogenous LOCCS								
Group	A			B			C		
Value	1	2	3	1	2	3	1	2	3
Firefly luciferase (RLU)	360	342	368	283	271	302	410	409	426
Renilla luciferase (RLU)	440	412	440	404	396	388	424	412	418
RLU	0.82	0.83	0.84	0.7	0.68	0.78	0.97	0.99	1.02
Average (mean ± SD)	0.83 ± 0.01*			0.72 ± 0.05*			0.99 ± 0.03		
	Exogenous LOCCS								
Group	D			E			F		
Value	1	2	3	1	2	3	1	2	3
Firefly luciferase (RLU)	143	135	131	275	279	302	287	296	289
Renilla luciferase (RLU)	340	392	364	376	360	412	396	360	376
RLU	0.42	0.34	0.36	0.73	0.78	0.73	0.72	0.82	0.77
Average (mean ± SD)	0.37 ± 0.04*			0.75 ± 0.03			0.77 ± 0.05		

*P < 0.05; *RLU* relative light unit

**Fig. 3:**
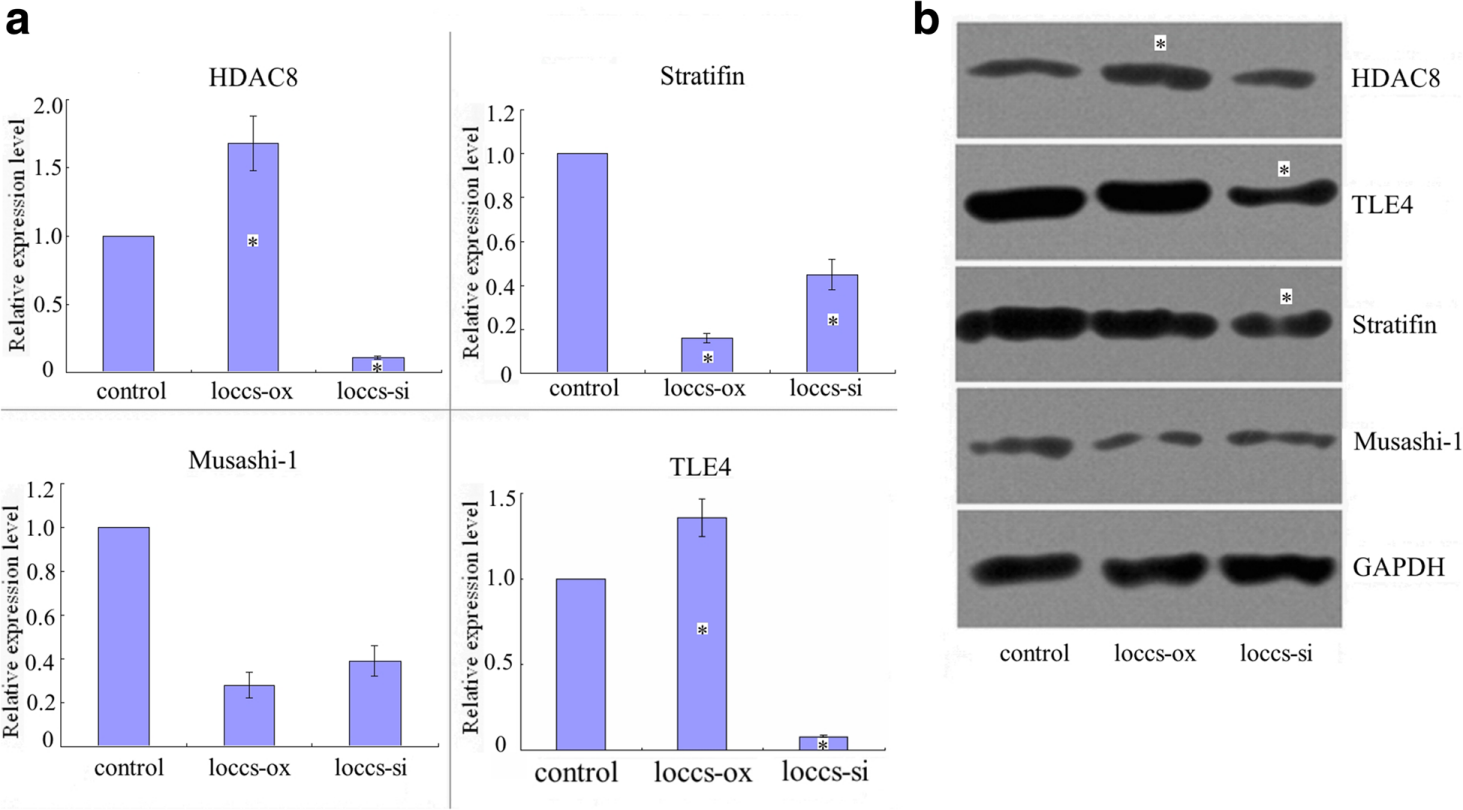
Expression of HDAC8, TLE4, stratifin, MSI1 mRNA and proteins in the process of knockdown or overexpression of LOCCS. **a** Expression of HDAC8, TLE4, stratifin and MSI1 mRNA assessed by quantitative PCR. **b** Cellular levels of the HDAC8, TLE4, stratifin and MSI1 proteins assessed by western blot. Each experiment was performed in triplicate. *P < 0.05

We next investigated the effects of up or downregulated expression of LOCCS on the expression levels of HDAC8, TLE4, stratifin and MSI1 mRNA (Fig. 3a) and protein (Fig. 3b). LOCCS up-regulated the expressions of HDAC8 and TLE4 mRNAs, and down-regulated that of stratifin mRNA. LOCCS may play an important regulatory role in their expressions and the specific mechanism needs further exploration.

### Knockdown of LOCCS suppresses CD133+/CD166+/CD44+ spheroid cells proliferation, invasion and migration in vitro and tumor growth in vivo

**Fig. 4:**
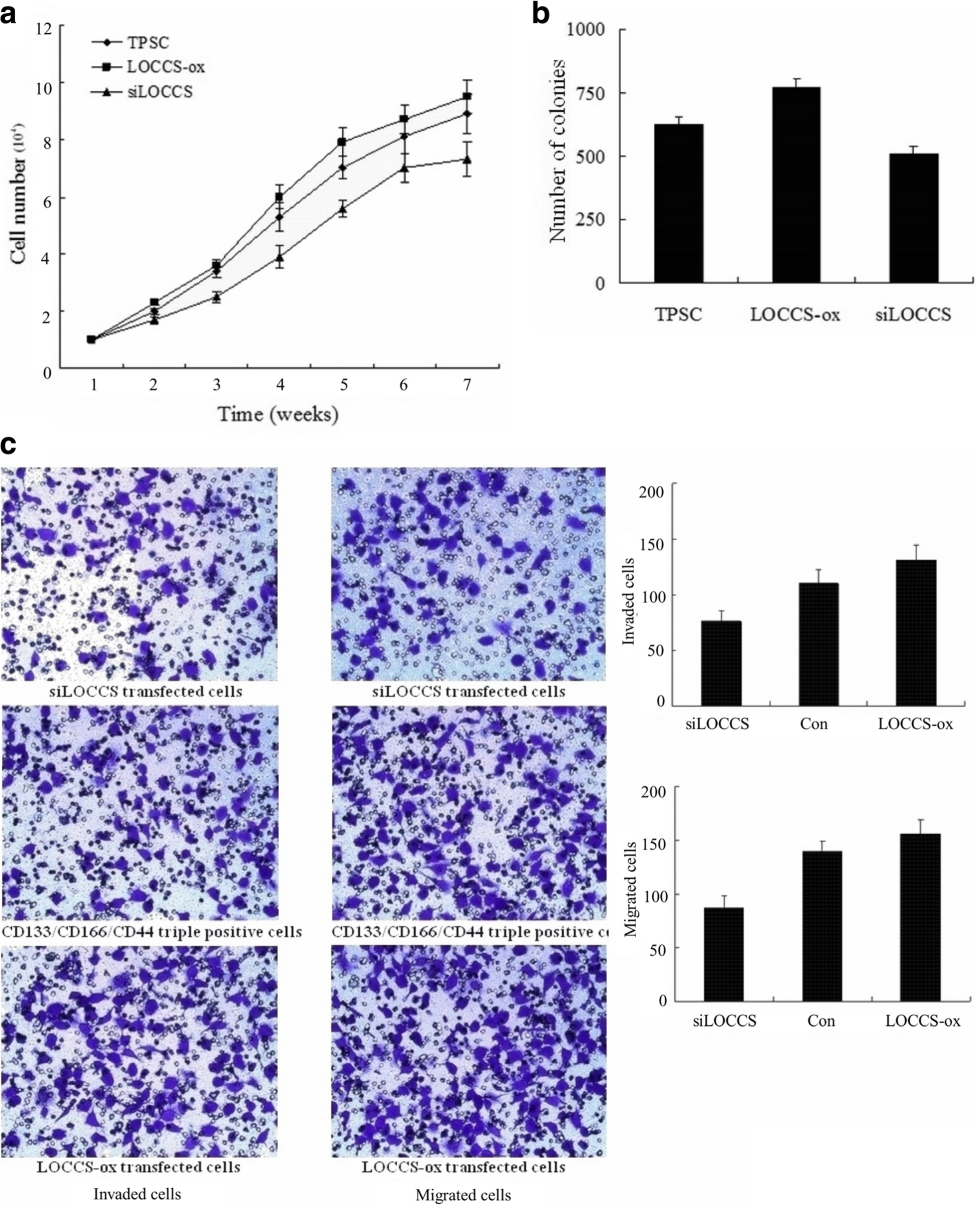
Knockdown or overexpression of LOCCS influences proliferation, invasion and migration of human colon cancer stem cells. **a** Growth curves of TPSC transfected with the siLOCCS or LOCCS-ox plasmid. **b** Colony formation rates of TPSC transfected with the siLOCCS or LOCCS-ox plasmid. **c** Invasion and migration of TPSC transfected with the siLOCCS or LOCCS-ox plasmid. Each experiment was performed in triplicate

To further identify the role of LOCCS, it was knocked down (siLOCCS) or overexpressed (LOCCS-ox) in TPSC. The cells were counted for 7 weeks after seeding and we found that the LOCCS-ox and siLOCCS cells have different growth curves (Fig. 4a). The siLOCCS cells grew relatively slowly especially after sex weeks. LOCCS-ox cells showed the fastest growth rate, whereas untransfected TPSC cells displayed an intermediate growth rate. The proliferation of the cells was then detected using coloning efficiency. TPSC and LOCCS-ox cells produced more numbers of spheres (625 ± 31 and 771 ± 38, respectively) than siLOCCS cells (508 ± 32) (*P* < 0.05) (Fig. 4b). Matrigel invasion and migration experiments showed a signifiant decrease of cell invasion and migration in siLOCCS-transfected group compared with the control and LOCCS-ox groups (*P* < 0.05) (Fig. 4c).

**Fig. 5:**
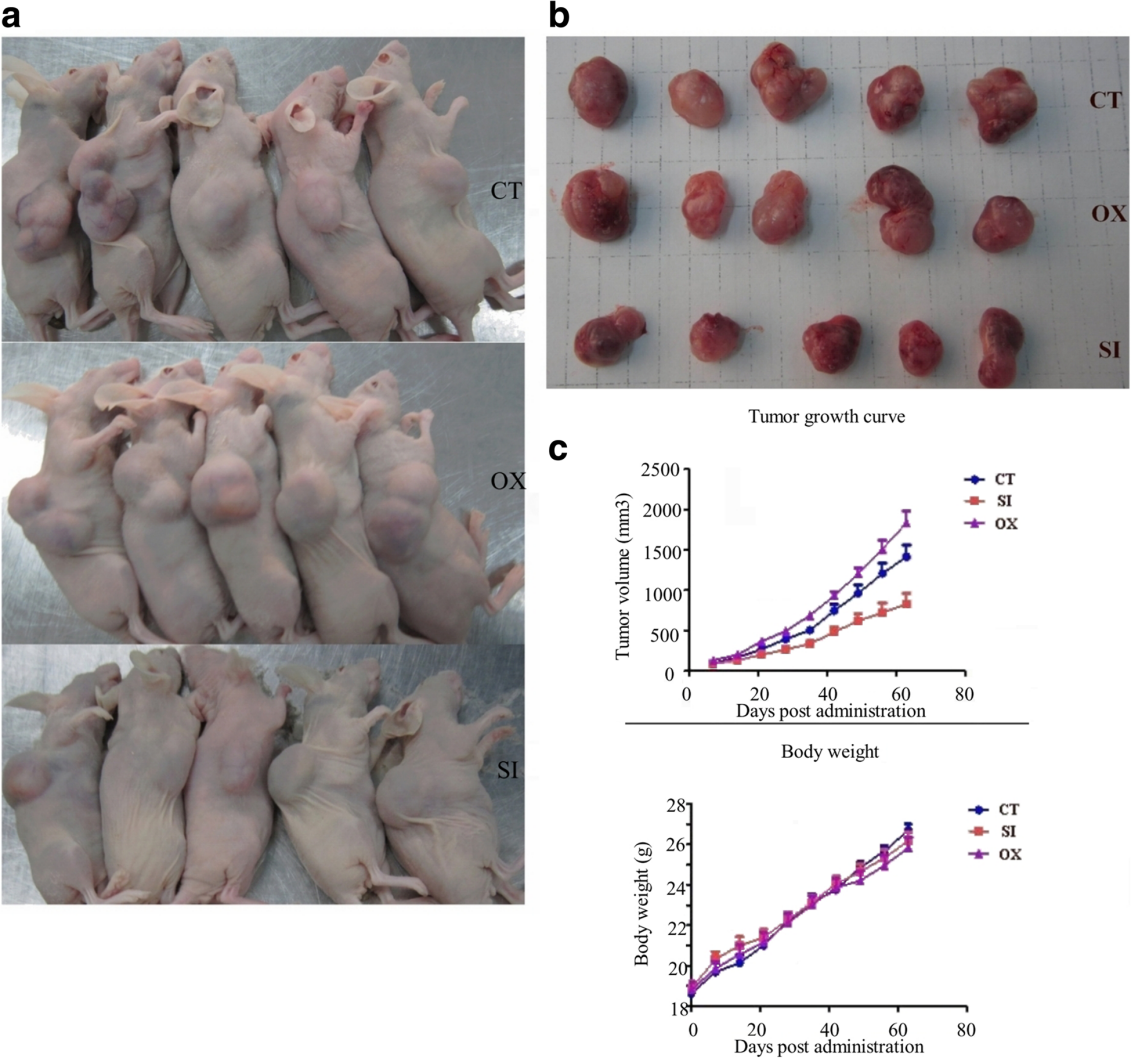
Knockdown or overexpression of LOCCS affects tumor growth of colon cancer stem cells in nude mice. **a** Representative tumor growth in nude mice 63 days after they were injected with 1 × 10^5^ TPSC transfected with the siLOCCS or LOCCS-ox plasmid. **b** Representative tumors dissected from the tumor-bearing mice 63 days after the mice were injected with 1 × 10^5^ TPSC transfected with the siLOCCS or LOCCS-ox plasmid. **c** Growth curves of tumors in the nude mice and the body weight of the nude mice throughout the 63-day observation period. CT: control group; OX: LOCCS-ox plasmid-transfected group; SI: siLOCCS plasmid-transfected group

The xenograft tumor experiment revealed that the tumor spheroids had high tumorigenicity. 10^4^ colon cancer sphere cells could induce visible tumors, whereas the same number of primary cultured cells failed to produce visible tumors. The result showed the tumor spheroids were enriched in CSCs. Growth rates of the TPSC, LOCCS-ox, and siLOCCS cells and xenografts and tumor sizes of the three groups at 63 days are shown in Fig. 5.

### MiR-93 and MSI1 mediated the tumor-suppressive effects of LOCCS knockdown on CD133+/CD166+/CD44+ spheroid cells

**Fig. 6:**
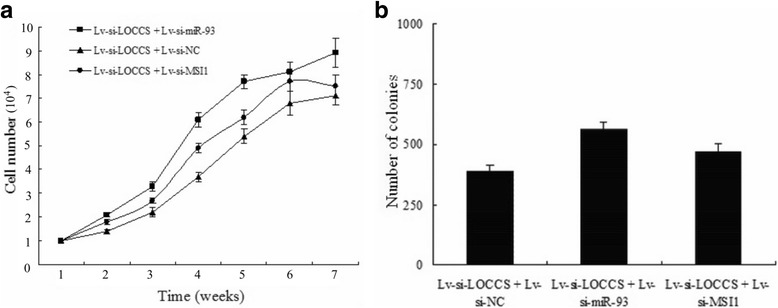
MiR-93 and MSI1 mediated the tumor-suppressive effects of LOCCS knockdown on TPSC. **a** Growth curves of TPSC transfected with the Lv-si-LOCCS + Lv-si-miR-93 or Lv-si-LOCCS + Lv-si-MSI1 plasmids. **b** Colony formation rates of TPSC transfected with the Lv-si-LOCCS + Lv-si-miR-93 or Lv-si-LOCCS + Lv-si-MSI1 plasmids. Each experiment was performed in triplicate

To determine whether the tumor inhibition of LOCCS knockdown were mediated by miR-93, miR-93 upregulation by LOCCS knockdown was rescued using Lv-si-miR-93 transfection before the evaluation of cell proliferation. Trypan blue and colony formation assays showed that the growth of TPSC in the Lv-si-LOCCS + Lv-si-miR-93 group was increased compared with the Lv-si-LOCCS + Lv-si-NC (control) group. In the Lv-si-LOCCS + Lv-si-miR-93 group, Lv-si-miR-93 rescued the suppression of Lv-si-LOCCS on cell growth (Fig. 6). This result suggested that miR-93 mediates the suppressive effects of LOCCS knockdown on colon cancer stem cell proliferation. Knockdown of MSI1 produced similar results to the above. In the Lv-si-LOCCS + Lv-si-MSI1 group, Lv-si-MSI1 also rescued the suppression of Lv-si-LOCCS on cell proliferation (Fig. 6).

## Discussion

Identification and localization of CR-CSCs remains difficult owing to the lack of widely accepted cancer stem cell markers. Isolation and identification of CR-CSCs can be achieved based on many cell-surface markers, such as CD133, CD166, CD24, CD44, beta1 integrin/CD29, ALDH-1, Lgr5, DCAMLK1, MSI1 and EpCAM [21, 22]. CD133, CD166 and CD44 are the important cell-surface markers which have recently been related to CR-CSCs.

CD133 is a 120-kDa five-transmembrane domain glycoprotein, which express on neural, endothelial, normal primitive hematopoietic and epithelial cells [23]. In recent years, it has been regard as a CSC surface marker for brain tumors [24] and colon [2], pancreatic [25], liver [26], and prostate [27] cancers. CD166 plays a key role in T-cell activation and proliferation, angiogenesis, hematopoiesis and axon fasciculation [28]. Some researches have confirmed that CD166 enriches CSC-like cells in various cancers [29, 30]. CD44 is an adhesion molecule which involves in many signal pathways [31]. This protein is also considered a stem-cell marker for cancers of the breast [32], pancreas [25], prostate [27], and colon [33] and for head and neck carcinoma [34]. High levels of the intestinal stem-cell marker MSI1 have been observed in CD44^+^ colon polyp cells [35].

In our research, we found that there was no obvious difference of CD133 expression in the adherent and spheroid cells isolated from human colon cancer tissue. However, the proportions of CD166^+^ and CD44^+^ cells in adherent cells were both much smaller than in spheroid cells. In other words, the levels of CD166 and CD44 were much higher in colon cancer spheroid cells. Other recent studies have found that CD44^+^ CRC cells display high tumorigenicity, especially combining CD133^+^ cells, whereas CD44^−^ cells do not form new tumors. Furthermore, CD44 can also be used in combination with CD166. A recent research showed that CD44^+^CD166^+^ colon cancer cells have greater ability to form xenografts in nude mice than CD44^+^CD166^−^, CD44^−^CD166^+^ or CD44^−^CD166^−^ cells [36, 37].

In the present study, the CD133^+^ subpopulations in spheroid cells were analyzed for parallel expression of CD166 and CD44. Most cells expressed only CD133 molecule and a mean of 1.09% of the cells were triple positive (CD133/CD166/CD44). The levels of CD133, CD166 and CD44 in spheroid cells was remarkable higher than in primary colon cancer cells, suggesting that the three molecules are mainly existed in undifferentiated tumor cells. We considered that a small amount of CSCs were present in primary cultured cells. They had the ability to form spheres and maintain an undifferentiated state. CD133^+^/CD166^+^/CD44^+^ spheroid cells possessed some characteristics as CSCs, including the ability to both proliferate and generate differentiated progeny, to resist chemotherapeutic drugs, and to produce xenografts in nude mice. We reveal that this marker combination (CD133^+^/CD166^+^/CD44^+^) may be very useful in the identification of colon CSCs.

Recently, LncRNAs are considered as key modulators in CSC biology. For example, Jiao et al. [12] revealed that MALAT-1 acted as an oncogenic lncRNA in carcinoma of pancreas, and regulated CSC marker expression. MALAT-1 also increases ratio of pancreatic CSCs and multidrug resistance, maintains self-renewing capacity, and accelerates tumor angiogenesis. Wang et al. [13] confirmed that the expression of lncRNA HOTAIR in ovarian cancer tissues and SKOV3 CD117^+^CD44^+^ CSCs increased obviously. In CD117^+^CD44^+^ CSCs, tumor growth and metastasis were significantly inhibited by downregulation HOTAIR expression.

Substantial evidence also confirms the important roles of miRNAs in regulating CSC biology. There are 11 up-regulated and 8 down-regulated miRNAs in CD133^+^ colon CSCs. These miRNAs have been observed to be associated with self-renewal and differentiation [16, 38]. In EpCAM-positive hepatocellular CSC, miR-181 was found to regulate differentiation by binding CDX2 and GATA6 [39]. In pancreatic carcinoma, CSCs show a signature of 210 miRNAs associated with proliferation and differentiation [40]. These researches show that miRNAs have an important role in regulating CSC proliferation, differentiation, and tumorigenesis. In our previous studies, we also found 46 dysregulated miRNAs in SW1116csc cells, 35 of which are overexpressed and 11 of which are downregulated. The downregulated miRNAs include miR-93 (16.7 times lower), and the upregulated expression level of miR-93 significantly inhibits cell growth and coloning efficiency of colon CSCs by negatively regulating mRNA and protein expression of HDAC8 and TLE4.

Dynamic expression of lncRNAs is involved in human carcinogenesis [41]. Considering the multiple targets of miRNAs, we hypothesized that there may be other lncRNAs as competing endogenous RNAs to regulate expression of key genes in CSCs. Competing endogenous RNA can act as a “sponge” to sequester miRNAs and therefore protect their target mRNAs from degradation [19, 20]. In the former research, we found that miR-93 acts as a cancer suppressor in CSCs by targeting the HDAC8 and TLE4 genes [16]. Searching for lncRNAs with an miR-93 binding site, we found LOCCS. We hypothesized that LOCCS may act as a competitive RNA for miR-93 in CSCs. In this study, we revealed that knockdown of LOCCS induced the upregulation of miR-93. Using bioinformatics analysis and luciferase reporter assays, we elucidated the direct binding site of miR-93 in LOCCS.

Further, we examined the effects of LOCCS knockdown on the biological behaviors of colon CSCs and showed that knockdown of LOCCS suppressed colon CSC proliferation, invasion and migration. The results revealed that knockdown of LOCCS had tumor inhibitory effects in colon CSCs. Furthermore, the in vivo results also confirmed that knockdown of LOCCS inhibited tumor proliferation, elucidating that LOCCS down-regulation could be potentially applied in clinical colon cancer therapy.

The molecular mechanisms underlying LOCCS actions in colon CSCs remains unknown. LOCCS may be required for maintenance of the self-renewal state and the suppression of the specific genes associated with lineage differentiation. This hypothesis is supported by our study elucidating that LOCCS serves as a competitive endogenous RNA (sponge) for miR-93, thus releasing miR-93 inhibition of target molecules, including MSI1, HDAC8 and TLE4, in CSCs. The MSI1 knockdown assay showed that depression of MSI1 rescued the inhibition of Lv-si-LOCCS on cell proliferation, invasion and migration. The data suggests that the tumor inhibitory effects of LOCCS knockdown are also mediated by MSI1 in TPSC.

As a RNA binding protein, MSI1 has important function to regulate proliferation and differentiation of stem or precursor cells [42]. It can inhibit translation of target mRNAs by binding to the 3′UTR of the target mRNA [43]. By downregulating APC, p21^WAF-1^ and NUMB, MSI1 positively regulates the Notch and Wnt signaling pathways [44–46]. It has recently become clear that MSI1 also binds to the 3′UTR of other mRNAs, which may involve in cell renewal, differentiation, apoptosis, and cell cycle control, and protein modification controlled by MSI1 have been identified [47]. From the above results, we conceive that LOCCS may regulate the expression of HDAC8 and TLE4 through miR-93, and it may also take part in the Notch and Wnt signaling pathways through MSI1. In this way, LOCCS may modulate colon cancer stem cell proliferation and differentiation, resistance to chemotherapeutic drugs, and ability to generate tumor xenografts.

## Conclusions

In the study, a new highly tumorigenic cell was identified from human colon adenocarcinomas. This kind of cell was isolated and purified using surface markers CD133, CD166, and CD44 and displayed some characteristics of stem cells. We have also shown for the first time that LOCCS expression is upregulated in colon CSCs. Knockdown of LOCCS reduced cell renewal, invasion and migration as well as reducing generation of tumor xenografts. Furthermore, miR-93 and MSI1 mediated the tumor suppression of LOCCS knockdown. There was reciprocal repression between LOCCS and miR-93 that mechanistic investigations suggested are attributable to direct binding of miR-93 by LOCCS. Taken together, our study elucidate that the lncRNA LOCCS may be a new modulator of human colon CSCs, which can exercise its functions by inhibiting miR-93 expression. Further researches of LOCCS may provide a new target for therapeutic strategies of colon cancer.

## Additional files

Additional file 1: Sequences of long intergenic non-protein-coding RNA 1567 (LOCCS) (DOC 27 kb)

Additional file 2: Sequences of pENTR/U6-Z1, pENTR/U6-Z2, pENTR/U6-Z3, pGL3M-miR-93, pcDNA-LOCCS and pcDNA-LOCCS-T plasmids (DOC 245 kb)

## Abbreviations

CRC: colorectal carcinoma
CR-CSCs: CRC stem cells
CSCs: cancer stem cells
FITC: fluorescein isothiocyanate
FLUC: firefly luciferase
GAPDH: glyceraldehyde-3-phosphate dehydrogenase
IC50: 50% reduction in cell viability
LINC01567: long intergenic non-protein-coding RNA 1567
lncRNAs: long non-coding RNAs
LOCCS: lncRNA overexpressed in colon cancer stem cells
miR-93: microRNA-93
MSI1: musashi-1
PBS: phosphate-buffered saline
PCC: primary cultured cells
PE: phycoerythrin
SFM: serum-free medium
siRNA: small interfering RNA
SSM: serum-supplemented medium
TPSC: triple positive spheroid cells
