# Supplementary material for: Long intergenic non-protein-coding RNA 1567 (LINC01567) acts as a “sponge” against microRNA-93 in regulating the proliferation and tumorigenesis of human colon cancer stem cells
Source: BMC Cancer. 2017 Nov 6;17:716. doi: 10.1186/s12885-017-3731-5 (PMC5674857; doi:10.1186/s12885-017-3731-5)
Supplement: Supplementary file 1 — Sequences of long intergenic non-protein-coding RNA 1567 (LOCCS) (DOC 27 kb) [file 12885_2017_3731_MOESM1_ESM.doc]

**LOCCS sequence**

**AGTGCTGTCGGAGGGTTGCTGGGGAAAGGAGATTGGCTGGCGAGGAAGATGAGGAATGTG**

**CTGCCAGGAGACTGGGATCACGGCCAGATGCTAATTACGATGGCCTGGGGGTGCTGGTAC**

**CCCCTGTCAGCATCATATCAGCCTCTCCACTTCAAATCC**

**TGCCCCTCTGATGTTAGAATCTGGCTGCAACCATCCCGTGATTCTGTGGAGCCTCGTGCC**

**TTACTTCTGCTGGCTTCTGTCTGTCGTCACTTCCGTTCTCCACCACCCTGGCCTCAATCA**

**AACACGTTTTCTCTGATGCAAAACTCGACCTTCATTTACAGAAAGGGTTATCTCAAGAGGCTTGGGA**

**TGGTTAAACCCTGCACATCTCAGAGGAAGGACTGGCTCTTCACCTGTTCCTGCAGGATAA**

**CTGCTAACGGCTTAAAATATTCTGCCTGATAAGAGGGCTTCTGTATATCTGGGGCCTTGG**

**GCCATGCCGGGTAGTTGATGCTAACAACATGATTTATGGTGGGTGCCTGCGGTCATGCTG**

**TATCAGCCTGACCTCTGGAGGGGCTGGACACTGGGTAACTGATCCCCAAGAAAAGCCATG**

**GACACTAAGGCTCAGGTGAGTTCCCCTGGTTGGCAGTGTTTTGCGTGTGTTGTCACACAT**

**CATTGCTGGGAGACTTAAGCACTGCACATATGACTATCTAGGAGGGTACAGCTGCAAGCT**

**CCTGCCTGGATTCTGCCCTGAGCACCTTTTTCCTTTGCTGATTTTGTTTATCTATTTATT**

**TATTTTTGAGATGGAGTCTCACTCTGTCACCAAGGTTGGAGTGCGGTGGCTGGATCTCGG**

**CTCATTGCAACCTCCACCTCCCAGGTTCACGTGATTCTTCTGCTTCAGCCTCCCAAGGAG**

**CTGGGATTACAGGTGCCAACCACCATGCCTGGCTAATTCTTATATGTTAGTAGAGACGGG**

**GTTTTGCCATGTTGGCCAGGCTGGTCTCGAACTCCTGAGCTCAAGTGACAAGGCCGCCGG**

**CCTCGACCTCCCAAAGTGCTGGGATTACAGGCATGAGCCACCGCGCCTGGCCCCTTTGCT**

**GATTTTTAATCCGCATCCTTCCACTGTGTTAAGCTGTAACTGTGAATACAACAGCTTTTT**

**TGAGTTCTGCGATTCTTTGCAGCAAATCGTCATACCTGAGCGTGTCTTGGGGACTCCCCG**

**ACACATCTTCCCACTCCCTCTTCTGTTTTCTCACTTCCTCTGGAGTAAACTGAAAGAGGC**

**CCCTGCTTGCCTCTTGCAAGGCAGTTCTGAACACACTGAGATCATTTGTGATCTCATATC**

**CTCTTCTAAGCAATTCATCAAGAAATTTCTTTCAAATAAACCCAGTGCTCTTCATGGAGG**

**TGATGCTGATGAAAATGACTTTCTCCAGCTTATTACACGTTTGCAGAAGCTCCTGTTTAA**

**GAGTTTGAGCATGTATGTGTGTGTGCATATACACCAGCACACACATGCATGCCCACAGCT**

**GTCATGCCTTCACCAGAACCAGGACGAGGAGTTATTCTATTGCCAGAATTGAAGAACCAT**

**TTCCACATATTGTAAGGTCAGTGAAAGTACAATGAAAAATACACCTGCTAGCATGCCTAT**

**GCATGGCCTCTCTGCAAATAAGATGAAGGTCCCTTCACCACCTGCTTGCACTGCTAATGG**

**CCCTGTAGATTCCACTGGATGGATTTGTAATTTTCTCAACCATTTCCCCTAATGCTGGAT**

**ATTTGGGTTATTTCCAACATTTTTACGCTTCCAAATAAGGCACCCATAATGATTCTTGCA**

**GAGTAATCATTAGGCTCTTCTATAATTATCTAATTATCCTTTTTTTTTTGTTTTTTTCTG**

**AGGCAGGGTCTTGCTCTGACACCCAGGCTGGAGTACAGCGGCACGATCATGGCTCACTGC**

**AGCCTCAGCCTCCCACGCTCAAGTGATTCCACAGTCTCAGCCTCCTGTGTAGCTGGGATC**

**ACAGGTGTGCGCCACCACGCCTGGCTAACTTTTTGTACTTTTTATAGAGACGGGGAGTTT**

**TTTTGTATTTTTTTAAATAGAGACGATGTCTCACCATGTTGCTTAGGCTGTTCTGGAACT**

**TCTGGACTCCAGTAATCCTCCTGCCTCAGCCTCCCAAGGTGCTGAGACTATAGGCATGAG**

**CCACCGTGCCCAGCCATATAATTATCTTAAGATAAATTTCTATACATGGAATTTTTATAC**

**GTGGAATTTCTATACATGAATTAAAGAGCACAGATTTTTGTATGTTGGTTGCTTGGTTTT**

**TAGAGGCAGTATCTCACTCCATTGCCCAGGTTGGAGTGCAGTGGTGCAATCATAGCTCAC**

**CGCACCACTGAATTCCTGGGCTCTAGGAGTCCTCCTTCCTCAGCCTCCTAAGCAACTAGG**

**ACTACAGAGTACATATGTTTTTAAGATCTTTGATATCTTATACCTTTTGGGGACTGTATC**

**AGTTAAAAACTCTACCAGTACTGTAGAAACATGATCGTGTCTCTGTTTCCTTGCCACACT**

**GGGCATTAATTACTTGATCCATTAATGAGTTAATTGCACCCTCAATAAGAGTATGGTAAA**

**GTGTGACACATTTACACTACAGAATATTATGCTGTAAAGAATAAGGATGTAGGCCAGGTG**

**AGGTGGCTCATGCCTGTAATCCCAGCACTTTGGGAGGCCGAGGCGGGCGGATGACGATGT**

**GAAGAGATCGAGACCATCCTGGCCAACATGTTGAAACCCCGTCTCTACTAACAGTACAAA**

**AATTAGCTGGGTGTGGTGGCAGGTGCCTGTAATTCCAGCTACTCGGGAGGCTGAGGCAGA**

**AGAATCGCTTGAACCTGGGAGGCGGAGGTTGCAGTGAGCCAAAATGGCGCTACTGCACTC**

**CAGACCGGGGACAGAGTGAGACTCTGTCTCCAAAACAAAAC**
