# Supplementary material for: Long intergenic non-protein-coding RNA 1567 (LINC01567) acts as a “sponge” against microRNA-93 in regulating the proliferation and tumorigenesis of human colon cancer stem cells
Source: BMC Cancer. 2017 Nov 6;17:716. doi: 10.1186/s12885-017-3731-5 (PMC5674857; doi:10.1186/s12885-017-3731-5)
Supplement: Supplementary file 2 — Sequences of pENTR/U6-Z1, pENTR/U6-Z2, pENTR/U6-Z3, pGL3M-miR-93, pcDNA-LOCCS and pcDNA-LOCCS-T plasmids (DOC 245 kb) [file 12885_2017_3731_MOESM2_ESM.doc]

shRNA primers：

| shRNA |  | sense | loop | antisense | RNA-polⅢ TTS |
| --- | --- | --- | --- | --- | --- |
| Z1-F(5’→3’) | CACC GGACGAGGAGTTATTCTATTG CGAA CAATAGAATAACTCCTCGTCC | | | | |
| Z1-R(3’→5’) | CCTGCTCCTCAATAAGATAAC GCTT GTTATCTTATTGAGGAGCAGG AAAA | | | | |
| Z2-F(5’→3’) | CACC GCCTCTCTGCAAATAAGATGA CGAA TCATCTTATTTGCAGAGAGGC | | | | |
| Z2-R(3’→5’) | CGGAGAGACGTTTATTCTACT GCTT AGTAGAATAAACGTCTCTCCG AAAA | | | | |
| Z3-F(5’→3’) | CACC GCTAATGGCCCTGTAGATTCC CGAA GGAATCTACAGGGCCATTAGC | | | | |
| Z3-R(3’→5’) | CGATTACCGGGACATCTAAGG GCTT CCTTAGATGTCCCGGTAATCG AAAA | | | | |

1.1 shRNA-Z1 and pENTR/U6-Z1

| Number | 10 20 30 40 50 60 70 80 |
| --- | --- |
| shRNA-Z1 | -------------------------------------------------------------------------------- |
| pENTR/U6-Z1 | GATGATTTTATTTTGACTGATAGTGACCTGTTCGTTGCAACAAATTGATGAGCAATGCTTTTTTATAATGCCAACTTTGT |
|  | 90 100 110 120 130 140 150 160 |
| shRNA-Z1 | -------------------------------------------------------------------------------- |
| pENTR/U6-Z1 | ACAAAAAAGCAGGCTTTAAAGGAACCAATTCAGTCGACTGGATCCGGTACCAAGGTCGGGCAGGAAGAGGGCCTATTTCC |
|  | 170 180 190 200 210 220 230 240 |
| shRNA-Z1 | -------------------------------------------------------------------------------- |
| pENTR/U6-Z1 | CATGATTCCTTCATATTTGCATATACGATACAAGGCTGTTAGAGAGATAATTAGAATTAATTTGACTGTAAACACAAAGA |
|  | 250 260 270 280 290 300 310 320 |
| shRNA-Z1 | -------------------------------------------------------------------------------- |
| pENTR/U6-Z1 | TATTAGTACAAAATACGTGACGTAGAAAGTAATAATTTCTTGGGTAGTTTGCAGTTTTAAAATTATGTTTTAAAATGGAC |
|  | 330 340 350 360 370 380 390 400 |
| shRNA-Z1 | -----------------------------------------------------------------------***CACCGGACG*** |
| pENTR/U6-Z1 | TATCATATGCTTACCGTAACTTGAAAGTATTTCGATTTCTTGGCTTTATATATCTTGTGGAAAGGACGAAA***CACCGGACG*** |
|  | 410 420 430 440 450 460 470 480 |
| shRNA-Z1 | ***AGGAGTTATTCTATTGCGAACAATAGAATAACTCCTCGTCC*** |
| pENTR/U6-Z1 | ***AGGAGTTATTCTATTGCGAACAATAGAATAACTCCTCGTCC***TTTTTTCTAGACCCAGCTTTCTTGTACAAAGTTGGCATT |
|  | 490 500 510 520 530 540 550 560 |
| shRNA-Z1 | -------------------------------------------------------------------------------- |
| pENTR/U6-Z1 | ATAAGAAAGCATTGCTTATCAATTTGTTGCAACGAACAGGTCACTATCAGTCAAAATAAAATCATTATTTGCCATCCAGC |
|  | 570 580 590 600 610 620 630 640 |
| shRNA-Z1 | -------------------------------------------------------------------------------- |
| pENTR/U6-Z1 | TGATATCCCCTATAGTGAGTCGTATTACATGGTCATAGCTGTTTCCTGGCAGCTCTGGCCCGTGTCTCAAAATCTCTGAT |
|  | 650 660 670 680 690 700 710 720 |
| shRNA-Z1 | -------------------------------------------------------------------------------- |
| pENTR/U6-Z1 | GTTACATTGCCCAAGATAAAAATATATCATCATGAAGAATAAAACTGTCTGCTTACATAAACAGTAATACAAGGGGTGTT |
|  | 730 740 750 760 770 780 790 800 |
| shRNA-Z1 | -------------------------------------------------------------------------------- |
| pENTR/U6-Z1 | ATGAGCCATATTCAACGCGAAACGTCGAGGCCGCGATTAAATTCCAACATGGATGCTGATTTATATGGATATAAATGGGC |
|  | 810 820 830 840 850 860 870 880 |
| shRNA-Z1 | -------------------------------------------------------------------------------- |
| pENTR/U6-Z1 | TCGCGATAATGTCGCGGCAATCAGGTGCGACAATCTATCGCTTGTATCGGGAAGGCCCGATGCGCCAGAGTGTTTCTGAA |
|  | 890 900 910 920 930 940 950 960 |
| shRNA-Z1 | -------------------------------------------------------------------------------- |
| pENTR/U6-Z1 | ACATTGATAAAGGTAGCCGTTGGCAAATGATCGCTACAGATGAGATGGCCAGACTAAACTGGGTTGACGACATTTAATGC |
|  | 970 |
| shRNA-Z1 | -------------- |
| pENTR/U6-Z1 | CTCTTCCGACATCC |

1.2 shRNA-Z2 and pENTR/U6-Z2

| Number | 10 20 30 40 50 60 70 80 |
| --- | --- |
| shRNA-Z2 | -------------------------------------------------------------------------------- |
| pENTR/U6-Z2 | GCACTCATATGATTTTATTTTGACTGATAGTGACCTGTTCGTTGCAACAAATTGATGAGCAATGCTTTTTTATAATGCCA |
|  | 90 100 110 120 130 140 150 160 |
| shRNA-Z2 | -------------------------------------------------------------------------------- |
| pENTR/U6-Z2 | ACTTTGTACAAAAAAGCAGGCTTTAAAGGAACCAATTCAGTCGACTGGATCCGGTACCAAGGTCGGGCAGGAAGAGGGCC |
|  | 170 180 190 200 210 220 230 240 |
| shRNA-Z2 | -------------------------------------------------------------------------------- |
| pENTR/U6-Z2 | TATTTCCCATGATTCCTTCATATTTGCATATACGATACAAGGCTGTTAGAGAGATAATTAGAATTAATTTGACTGTAAAC |
|  | 250 260 270 280 290 300 310 320 |
| shRNA-Z2 | -------------------------------------------------------------------------------- |
| pENTR/U6-Z2 | ACAAAGATATTAGTACAAAATACGTGACGTAGAAAGTAATAATTTCTTGGGTAGTTTGCAGTTTTAAAATTATGTTTTAA |
|  | 330 340 350 360 370 380 390 400 |
| shRNA-Z2 | ------------------------------------------------------------------------------***CA*** |
| pENTR/U6-Z2 | AATGGACTATCATATGCTTACCGTAACTTGAAAGTATTTCGATTTCTTGGCTTTATATATCTTGTGGAAAGGACGAAA***CA*** |
|  | 410 420 430 440 450 460 470 480 |
| shRNA-Z2 | ***CCGCCTCTCTGCAAATAAGATGACGAATCATCTTATTTGCAGAGAGGC***-------------------------------- |
| pENTR/U6-Z2 | ***CCGCCTCTCTGCAAATAAGATGACGAATCATCTTATTTGCAGAGAGGC***TTTTTTCTAGACCCAGCTTTCTTGTACAAAGT |
|  | 490 500 510 520 530 540 550 560 |
| shRNA-Z2 | -------------------------------------------------------------------------------- |
| pENTR/U6-Z2 | TGGCATTATAAGAAAGCATTGCTTATCAATTTGTTGCAACGAACAGGTCACTATCAGTCAAAATAAAATCATTATTTGCC |
|  | 570 580 590 600 610 620 630 640 |
| shRNA-Z2 | -------------------------------------------------------------------------------- |
| pENTR/U6-Z2 | ATCCAGCTGATATCCCCTATAGTGAGTCGTATTACATGGTCATAGCTGTTTCCTGGCAGCTCTGGCCCGTGTCTCAAAAT |
|  | 650 660 670 680 690 700 710 720 |
| shRNA-Z2 | -------------------------------------------------------------------------------- |
| pENTR/U6-Z2 | CTCTGATGTTACATTGCACAAGATAAAAATATATCATCATGAACAATAAAACTGTCTGCTTACATAAACAGTAATACAAG |
|  | 730 740 750 760 770 780 790 800 |
| shRNA-Z2 | -------------------------------------------------------------------------------- |
| pENTR/U6-Z2 | GGGTGTTATGAGCCATATTCAACGGGAAACGTCGAGGCCGCGATTAAATTCCAACATGGATGCTGATTTATATGGGTATA |
|  | 810 820 830 840 850 860 870 880 |
| shRNA-Z2 | -------------------------------------------------------------------------------- |
| pENTR/U6-Z2 | AATGGGCTCGCGATAATGTCGGGCAATCAGGTGCGACAATCTATCGCTTGTATGGGAAGCCCGATGCGCCAGAGTTGTTT |
|  | 890 900 910 920 930 940 950 960 |
| shRNA-Z2 | -------------------------------------------------------------------------------- |
| pENTR/U6-Z2 | CTGAAACATGGCAAAGGTAGCGTTGCCAATGATGTTACAGATGAGATGGTCAGACTAAACTGGCTGACGGAATTTATGCC |
|  | 970 980 990 |
| shRNA-Z2 | ------------------------------ |
| pENTR/U6-Z2 | TCCTTCCGACCATCAAGCATTTTATCCGTA |

1.3 shRNA-Z3 and pENTR/U6-Z3

| Number | 10 20 30 40 50 60 70 80 |
| --- | --- |
| shRNA-Z3 | -------------------------------------------------------------------------------- |
| pENTR/U6-Z3 | TCACTATGATTTTATTTTGACTGATAGTGACCTGTTCGTTGCAACAAATTGATGAGCAATGCTTTTTTATAATGCCAACT |
|  | 90 100 110 120 130 140 150 160 |
| shRNA-Z3 | -------------------------------------------------------------------------------- |
| pENTR/U6-Z3 | TTGTACAAAAAAGCAGGCTTTAAAGGAACCAATTCAGTCGACTGGATCCGGTACCAAGGTCGGGCAGGAAGAGGGCCTAT |
|  | 170 180 190 200 210 220 230 240 |
| shRNA-Z3 | -------------------------------------------------------------------------------- |
| pENTR/U6-Z3 | TTCCCATGATTCCTTCATATTTGCATATACGATACAAGGCTGTTAGAGAGATAATTAGAATTAATTTGACTGTAAACACA |
|  | 250 260 270 280 290 300 310 320 |
| shRNA-Z3 | -------------------------------------------------------------------------------- |
| pENTR/U6-Z3 | AAGATATTAGTACAAAATACGTGACGTAGAAAGTAATAATTTCTTGGGTAGTTTGCAGTTTTAAAATTATGTTTTAAAAT |
|  | 330 340 350 360 370 380 390 400 |
| shRNA-Z3 | ---------------------------------------------------------------------------***CACCG*** |
| pENTR/U6-Z3 | GGACTATCATATGCTTACCGTAACTTGAAAGTATTTCGATTTCTTGGCTTTATATATCTTGTGGAAAGGACGAAA***CACCG*** |
|  | 410 420 430 440 450 460 470 480 |
| shRNA-Z3 | ***CTAATGGCCCTGTAGATTCCCGAAGGAATCTACAGGGCCATTAGC***----------------------------------- |
| pENTR/U6-Z3 | ***CTAATGGCCCTGTAGATTCCCGAAGGAATCTACAGGGCCATTAGC***TTTTTTCTAGACCCAGCTTTCTTGTACAAAGTTGG |
|  | 490 500 510 520 530 540 550 560 |
| shRNA-Z3 | -------------------------------------------------------------------------------- |
| pENTR/U6-Z3 | CATTATAAGAAAGCATTGCTTATCAATTTGTTGCAACGAACAGGTCACTATCAGTCAAAATAAAATCATTATTTGCCATC |
|  | 570 580 590 600 610 620 630 640 |
| shRNA-Z3 | -------------------------------------------------------------------------------- |
| pENTR/U6-Z3 | CAGCTGATATCCCCTATAGTGAGTCGTATTACATGGTCATAGCTGTTTCCTGGCAGCTCTGGCCCGTGTCTCAAAATCTC |
|  | 650 660 670 680 690 700 710 720 |
| shRNA-Z3 | -------------------------------------------------------------------------------- |
| pENTR/U6-Z3 | TGATGTTACATTGCACAAGATAAAAATATATCATCATGAACAATAAAACTGTCTGCTTACATAAACAGTAATACAAGGGG |
|  | 730 740 750 760 770 780 790 800 |
| shRNA-Z3 | -------------------------------------------------------------------------------- |
| pENTR/U6-Z3 | TGTTATGAGCCATATTCAACGGGAAACGTCGAGGCCGCGATTAAATTCCAACATGGATGCTGATTTATATGGGTATAAAT |
|  | 810 820 830 840 850 860 870 880 |
| shRNA-Z3 | -------------------------------------------------------------------------------- |
| pENTR/U6-Z3 | GGGCTCGCGATAATGTCGGGCAATCAGGTGCGACAATCTATCGCTTGTATGGGAAGCCCGATGCGCCAGAGTTGTTTCTG |
|  | 890 900 910 920 930 940 950 960 |
| shRNA-Z3 | -------------------------------------------------------------------------------- |
| pENTR/U6-Z3 | AAACATGGCAAAGGTAGCGTTGCCAATGATGTTACAGATGAGATGGTCAGACTAAACTGGCTGACGGAATTTATGCCTTC |
|  | 970 980 990 1000 1010 1020 1030 1040 |
| shRNA-Z3 | -------------------------------------------------------------------------------- |
| pENTR/U6-Z3 | TTCCGACCATCAAGCATTTTATCCGTACTCCTGATGATGCATGGTACTCACCACTGGCGATCCCCGAAATCAGCATTCCA |
|  | 1050 |
| shRNA-Z3 | ---------- |
| pENTR/U6-Z3 | GGTATTAGAG |

**pGL3M-miR-93 plasmid DNA sequences**

| Number | 10 20 30 40 50 60 70 80 |
| --- | --- |
| pre-miR-93-R | -------------------------------------------------------------------------------- |
| pGL3M-miR-93 | AACACCGCGTCTTCGGCTCGAGATTCGAGCAGCTCTAGACCGGGGGCTCGGGAAGTGCTAGCTCAGCAGTAGGTTGGGAA |
|  | 90 100 110 120 130 140 150 160 |
| pre-miR-93-R | -----------------------------------------------------------***CCGGGGGCTCGGGAAGTGCTA*** |
| pGL3M-miR-93 | TCACACTACCTGCACGAACAGCACTTTGGAGCCCCCAGTCTAGAGTCGAGCACTCTAGA***CCGGGGGCTCGGGAAGTGCTA*** |
|  | 170 180 190 200 210 220 230 240 |
| pre-miR-93-R | ***GCTCAGCAGTAGGTTGGGTAATCACACTACCTGCACGAACAGCACTTTGGAGCCCCCAG***--------------------- |
| pGL3M-miR-93 | ***GCTCAGCAGTAGGTTGGGTAATCACACTACCTGCACGAACAGCACTTTGGAGCCCCCAG***TCTAGAGTCGAGCAAATCTCT |
|  | 250 260 270 280 290 300 310 320 |
| pre-miR-93-R | -------------------------------------------------------------------------------- |
| pGL3M-miR-93 | AGAGGATCCCCGGGTACCGAGCTCGAATTCGTAATCATGGTCATAGCTGTTTCCTGTGTGAAATTGTTATCCGCTCACAA |
|  | 330 340 350 360 370 380 390 400 |
| pre-miR-93-R | -------------------------------------------------------------------------------- |
| pGL3M-miR-93 | TTCCACACAACATACGAGCCGGAAGCATAAAGTGTAAAGCCTGGGGTGCCTAATGAGTGAGCTAACTCACATTAATTGCG |
|  | 410 420 430 440 450 460 470 480 |
| pre-miR-93-R | -------------------------------------------------------------------------------- |
| pGL3M-miR-93 | TTGCGCTCACTGCCCGCTTTCCAGTCGGGAAACCTGTCGTGCCAGCTGCATTAATGAATCGGCCAACGCGCGGGGAGAGG |
|  | 490 500 510 520 530 540 550 560 |
| pre-miR-93-R | -------------------------------------------------------------------------------- |
| pGL3M-miR-93 | CGGTTCCCCCTATTGGGCGCTCTTCCGCTTCCTCGCTCACTGACTCGCTGCGCTCGGTCGTTCGGCTGCGGCGAGCGGTA |
|  | 570 580 590 600 610 620 630 640 |
| pre-miR-93-R | -------------------------------------------------------------------------------- |
| pGL3M-miR-93 | TCAGCTCACTCAAAGGCGGTAATACGGTTATCCCACAGAATCAGGGGATAACGCAGGAAAGAACATGTGAGCAAAAGGGC |
|  | 650 660 670 680 690 700 710 720 |
| pre-miR-93-R | --- |
| pGL3M-miR-93 | CAG |

**pcDNA-LOCCS plasmid DNA sequences**

| Number | 10 20 30 40 50 60 70 80 |
| --- | --- |
| miR-93match | -------------------------------------------------------------------------------- |
| LOCCS | TACATGATTACGATTCGAGCTCGGTACCCGGGGATCCTCTAGAGATTTATGTTAGTAGAGACGGGGTTTTGCCATGTTGG |
|  | 90 100 110 120 130 140 150 160 |
| miR-93 | --------------------------------------------------------***CAAAGTGCTG***TTCG***T***G***CAGG***T***A***G |
| LOCCS | CCAGGCTGGTCTCGAACTCCTGAGCTCAAGTGACAAGGCCGCCGGCCTCGACCTCC***CAAAGTGCTG***GGAT***T***A***CAGG***C***A***TG |
|  | 170 180 190 200 210 220 230 240 |
| miR-93 | -------------------------------------------------------------------------------- |
| LOCCS | AGCCACCGCGCCTGGCCCCTTTGCTGATTTTTAATCCGCATCCTTCCACTGTGTTAAGCTGTAACTGTGAATACAACAGC |
|  | 250 260 270 280 290 300 310 320 |
| miR-93 | -------------------------------------------------------------------------------- |
| LOCCS | TTTTTTGAGTTCTGCGATTCTTTGCAGCAAATCGTCATACCTGAGCAATCGTCGACCTGCAGGCATGCAAGCTTGGCACT |
|  | 330 340 350 360 370 380 390 400 |
| miR-93 | -------------------------------------------------------------------------------- |
| LOCCS | GGCCGTCGTTTTACAACGTCGTGACTGGGAAAACCCTGGCGTTACCCAACTTAATCGCCTTGCAGCACATCCCCCTTTCG |
|  | 410 420 430 440 450 460 470 480 |
| miR-93 | -------------------------------------------------------------------------------- |
| LOCCS | CCAGCTGGCGTAATAGCGAAGAGGCCCGCACCGATCGCCCTTCCCAACAGTTGCGCAGCCTGAATGGCGAATGGCGCCTG |
|  | 490 500 510 520 530 540 550 560 |
| miR-93 | -------------------------------------------------------------------------------- |
| LOCCS | ATGCGGTATTTTCTCCTTACGCATCTGTGCGGTATTTCACACCGCATATGGTGCACTCTCAGTACAATCTGCTCTGATGC |
|  | 570 580 590 600 610 620 630 640 |
| miR-93 | -------------------------------------------------------------------------------- |
| LOCCS | CGCATAGTTAAGCCAGCCCCGACACCCGCCAACACCCGCTGACGCGCCCTGACGGGCTTGTCTGCTCCCGGCATCCGCTT |
|  | 650 660 670 680 690 700 710 720 |
| miR-93 | -------------------------------------------------------------------------------- |
| LOCCS | ACAGACAAGCTGTGACCGTCTCCGGGAGCTGCATGTGTCAGAGGTTTTCACCGTCATCACCGAAACGCGCGAGACGAAAG |
|  | 730 740 750 760 770 780 790 800 |
| miR-93 | -------------------------------------------------------------------------------- |
| LOCCS | GGCCTCGTGATACGCCTATTTTTATAGGTTAATGTCATGATAATAATGGTTTCTTAGACGTCAGGTGGCACTTTTCGGGG |
|  | 810 820 830 840 850 860 870 880 |
| miR-93 | -------------------------------------------------------------------------------- |
| LOCCS | AAATGTGCGCGGAACCCCTATTTGTTTATTTTTCTAAATACATTCAAATATGTATCCGCTCATGAGACAATAACCCTGAT |
|  | 890 900 910 920 930 940 950 960 |
| miR-93 | ---------------------------------------------------------------------------- |
| LOCCS | AAATGCTTCAATAATATTGAAAAAGGAAGAGTATGAGTATTCAACATTTCCGTGTCGCCCTTATTCCCTTTTTTGC |

**pcDNA-LOCCS-T plasmid DNA sequences**

| Number | 10 20 30 40 50 60 70 80 |
| --- | --- |
| LOCCS-T | ---------------***CGAGCTCGGTACCCGGGGATCCTCTAGAGATTTATGTTAGTAGAGACGGGGTTTTGCCATGTTGG*** |
| LOCCS | TACATGATTACGATT***CGAGCTCGGTACCCGGGGATCCTCTAGAGATTTATGTTAGTAGAGACGGGGTTTTGCCATGTTGG*** |
|  | 90 100 110 120 130 140 150 160 |
| LOCCS-T | ***CCAGGCTGGTCTCGAACTCCTGAGCTCAAGTGACAAGGCCGCCGGCCTCGACCTCCCA***TGT***G***A***G***A***T***CACGA***T***G***CA***A-***CAT*** |
| LOCCS | ***CCAGGCTGGTCTCGAACTCCTGAGCTCAAGTGACAAGGCCGCCGGCCTCGACCTCCCA***-AA***G***T***G***C***T***GGGAT***T***A***CA***GG***CAT*** |
|  | 170 180 190 200 210 220 230 240 |
| LOCCS-T | ***GAGCCACCGCGCCTGGCCCCTTTGCTGATTTTTAATCCGCATCCTTCCACTGTGTTAAGCTGTAACTGTGAATACAACAG*** |
| LOCCS | ***GAGCCACCGCGCCTGGCCCCTTTGCTGATTTTTAATCCGCATCCTTCCACTGTGTTAAGCTGTAACTGTGAATACAACAG*** |
|  | 250 260 270 280 290 300 310 320 |
| LOCCS-T | ***CTTTTTTGAGTTCTGCGATTCTTTGCAGCAAATCGTCATACCTGAGCAATCGTCGACCTGCAGGCATGCAAGCTTGGCAC*** |
| LOCCS | ***CTTTTTTGAGTTCTGCGATTCTTTGCAGCAAATCGTCATACCTGAGCAATCGTCGACCTGCAGGCATGCAAGCTTGGCAC*** |
|  | 330 340 350 360 370 380 390 400 |
| LOCCS-T | ***TGGCCGTCGTTTTACAACGTCGTGACTGGGAAAACCCTGGCGTTACCCAACTTAATCGCCTTGCAGCACATCCCCCTTTC*** |
| LOCCS | ***TGGCCGTCGTTTTACAACGTCGTGACTGGGAAAACCCTGGCGTTACCCAACTTAATCGCCTTGCAGCACATCCCCCTTTC*** |
|  | 410 420 430 440 450 460 470 480 |
| LOCCS-T | ***GCCAGCTGGCGTAATAGCGAAGAGGCCCGCACCGATCGCCCTTCCCAACAGTTGCGCAGCCTGAATGGCGAATGGCGCCT*** |
| LOCCS | ***GCCAGCTGGCGTAATAGCGAAGAGGCCCGCACCGATCGCCCTTCCCAACAGTTGCGCAGCCTGAATGGCGAATGGCGCCT*** |
|  | 490 500 510 520 530 540 550 560 |
| LOCCS-T | ***GATGCGGTATTTTCTCCTTACGCATCTGTGCGGTATTTCACACCGCATATGGTGCACTCTCAGTACAATCTGCTCTGATG*** |
| LOCCS | ***GATGCGGTATTTTCTCCTTACGCATCTGTGCGGTATTTCACACCGCATATGGTGCACTCTCAGTACAATCTGCTCTGATG*** |
|  | 570 580 590 600 610 620 630 640 |
| LOCCS-T | ***CCGCATAGTTAAGCCAGCCCCGACACCCGCCAACACCCGCTGACGCGCCCTGACGGGCTTGTCTGCTCCCGGCATCCGCT*** |
| LOCCS | ***CCGCATAGTTAAGCCAGCCCCGACACCCGCCAACACCCGCTGACGCGCCCTGACGGGCTTGTCTGCTCCCGGCATCCGCT*** |
|  | 650 660 670 680 690 700 710 720 |
| LOCCS-T | ***TACAGACAAGCTGTGACCGTCTCCGGGAGCTGCATGTGTCAGAGGTTTTCACCGTCATCACCGAAACGCGCGAGACGAAA*** |
| LOCCS | ***TACAGACAAGCTGTGACCGTCTCCGGGAGCTGCATGTGTCAGAGGTTTTCACCGTCATCACCGAAACGCGCGAGACGAAA*** |
|  | 730 740 750 760 770 780 790 800 |
| LOCCS-T | ***GGGCCTCGTGATACGCCTATTTTTATAGGTTAATGTCATGATAATAATGGTTTCTTAGACGTCAGGTGGCACTTTTCGGG*** |
| LOCCS | ***GGGCCTCGTGATACGCCTATTTTTATAGGTTAATGTCATGATAATAATGGTTTCTTAGACGTCAGGTGGCACTTTTCGGG*** |
|  | 810 820 830 840 850 860 870 880 |
| LOCCS-T | ***GAAATGTGCGCGGAACCCCTATTTGTTTATTTTTCTAAATACATTCAAATATGTATCCGCTCATGAGACAATAACCCTGA*** |
| LOCCS | ***GAAATGTGCGCGGAACCCCTATTTGTTTATTTTTCTAAATACATTCAAATATGTATCCGCTCATGAGACAATAACCCTGA*** |
|  | 890 900 910 920 930 940 950 960 |
| LOCCS-T | ***TAAATGCTTCAATAATATTGAAAAAGGAAGAGTATGAGTATTCAACATTTCCGTGTCGCCCTTATTC***---------- |
| LOCCS | ***TAAATGCTTCAATAATATTGAAAAAGGAAGAGTATGAGTATTCAACATTTCCGTGTCGCCCTTATTC***CCTTTTTTGC |
